# Community efficacy for non-communicable disease management (COEN): Conceptualization and measurement

**DOI:** 10.1371/journal.pgph.0003549

**Published:** 2024-08-14

**Authors:** Shangzhi Xiong, Gangjiao Zhu, Rahul Malhotra, Xinyue Chen, Enying Gong, Zhan Wang, Jian’An Zhang, Weixia Peng, Shiwei Wang, Xurui Jin, Nicholas Peoples, Truls Østbye, Maoyi Tian, Lijing L. Yan

**Affiliations:** 1 Global Health Research Centre, Duke Kunshan University, Kunshan, China; 2 The George Institute for Global Health, Faulty of Medicine and Health, University of New South Wales, Sydney, Australia; 3 School of Public Health, Wuhan University, Wuhan, China; 4 Health Services and Systems Research, Duke-NUS Medical School, Singapore, Singapore; 5 Center for Ageing Research and Education, Duke-NUS Medical School, Singapore, Singapore; 6 SingHealth-Duke-NUS Global Health Institute, Singapore, Singapore; 7 Department of International Health, Bloomberg School of Public Health, Johns Hopkins University, Baltimore, Maryland, United States of America; 8 School of Population Medicine and Public Health, China Academy of Medical Sciences & Peking Union Medical College, Beijing, China; 9 Department of Agricultural Economics, College of Agriculture, Purdue University, West Lafayette, Indiana, United States of America; 10 Taicang Disease Prevention and Control Centre, Taicang, China; 11 School of Public Health, Fudan University, Shanghai, China; 12 MindRank AI Ltd., Hangzhou, China; 13 Baylor College of Medicine, Houston, Texas, United States of America; 14 Duke Global Health Institute, Duke University, Durham, North Carolina, United States of America; 15 School of Public Health, Harbin Medical University, Harbin, China; 16 Department of Preventive Medicine, Feinberg School of Medicine, Northwestern University, Chicago, Illinois, United States of America; 17 The George Institute for Global Health, Beijing, China; University of Cape Town, SOUTH AFRICA

## Abstract

The importance of community-based non-communicable disease (NCD) management has been internationally recognized. However, currently, no instrument is available to evaluate a community’s ability to provide NCD management for its residents. This study defined such an ability as “Community Efficacy for NCD Management” (COEN), and aimed to conceptualize, develop and validate a scale to measure COEN. We first conducted literature review, expert interviews, and Delphi panels to conceptualize COEN and select scale items. Then, we conducted two rounds of community surveys and interviews to validate the COEN scale among local residents in three cities in China. We used Cronbach’s alpha to test the scale’s internal consistency, Kappa test for test-retest reliability, and exploratory factor analysis for structural validity. COEN was conceptualized as “the ability of a community to provide NCD management for its residents, reflected by its natural environment, social relationships, community resources, health services, and resident-engaging activities.” The first community research among 345 residents yielded a 38-item COEN scale with high internal consistency (Cronbach’s alpha = 0.86) and acceptable test-retest reliability (Kappa value >0.2). The second community research tested a shortened COEN scale among 657 residents, yielding a final COEN scale with 14 items from five factors: community management (n = 3), social relationships (n = 4), resource accessibility (n = 3), community health services (n = 2), and resident engagement (n = 2), with an overall Cronbach’s alpha of 0.79. COEN is a meaningful concept in contextualizing and evaluating NCD management anchored in the community, and the COEN scale is a multi-domain reliable tool to quantify COEN, which can be used to guide future related research and practice in public health.

## Introduction

Globally, communities are considered the cornerstone of efforts to achieve public health control of non-communicable diseases (NCDs)—a group of diseases that require continuous care with essentially life-long durations. The World Health Organization’s (WHO) “best buys” and recommendations for NCD prevention and control, for example, repeatedly emphasizes the importance of “communities” [1]. According to the literature, task-shifting from hospital-based physicians to community-based non-physician health providers, for instance, is effective and affordable for improving access to NCD care [2]. Studies have also investigated the effects of community-based interventions on behavioral change [3], peer-support [3, 4], and policy development for health promotion [3], such as community advocacy for salt substitution policies [5].

Over the past decades, many countries have prioritized strengthening the role of community in NCD management. A recent systematic review suggested that community-based interventions and health promotion programs were, in general, cost-effective for NCD control (e.g., reducing cardiovascular events and mortality) in East Asian countries [6]. In China, the central government launched a national public health strategy by engaging community health facilities in providing essential public health services [7, 8]. China’s community-based NCD management, however, still faces challenges. These include an insufficiently qualified workforce [9–11], regional resource disparities [10, 11], and residents’ lack of incentives for seeking community-based NCD care [9]. Additionally, most of the existing community-based NCD research and practices in China have focused on service delivery, with limited evidence concerning other strategies such as peer-support and participatory activities. A cohesive guiding theory may support strengthen the role of communities in NCD management in countries with such public health commitment.

Existing literature offers several relevant concepts and theories. “Community mobilization”, for example, is a capacity-building process for community members to improve conditions on a participatory and sustained basis [12]. “Community empowerment” involves a continuous process of shifting power relations between individuals and social groups [13]. In the context of public health, community empowerment has largely focused on environmental changes for improving health outcomes [13]. “Community engagement”, as another example, focuses on specific community-based programs, by engaging community members in identifying priorities, implementing and evaluating solutions, to ensure successful program rollout [14]. These existing concepts all describe “processes”—capacity building, environment changing, or program rollout, and none of them provides a measurable depiction of a community in improving people’s health. Moreover, none of them is tailored for NCD management.

In this study, we conceive of a new concept–“Community Efficacy for NCD management” (COEN)–which we define as “*the ability of a community to provide NCD management for its residents*”, as a tool to assist researchers, practitioners, and decision makers in evaluating and monitoring communities’ roles in NCD management, and subsequently to seek improvements. Distinguished from the aforementioned concepts such as community engagement and empowerment that describe community-based “processes”, COEN was constructed to reflect and quantify the “current status” (i.e., efficacy) of a community, specifically in NCD management. The two main objectives of this study were: (1) to conceptualize “COEN” in the context of communities in China, and (2) to develop and validate a scale for measuring “COEN”. The findings should provide theoretical support and practical guidance on strengthening community-based NCD management.

## Methods

### Study design

**Fig 1:**
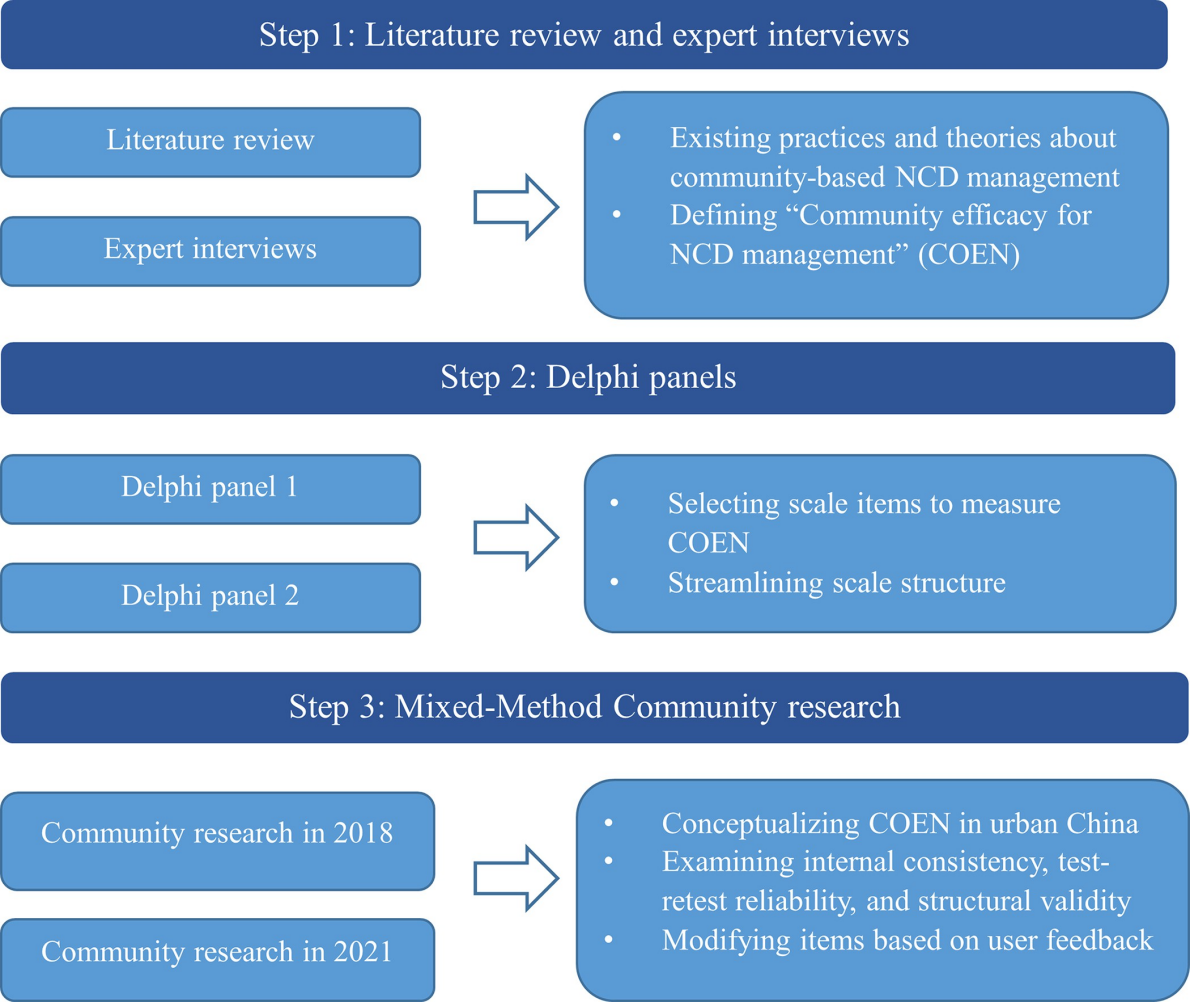
Three-step process of the study design.

We used a three-step process for the conceptualization of COEN and development and validation of the COEN scale (Fig 1). First, we conducted literature review and expert interviews to explore existing practices and theories about community-based NCD management globally and in China. Second, we conducted Delphi panels to generate items of the COEN scale, streamline the scale structure, and examine the face validity. These two steps have been published elsewhere respectively [15, 16], and more details about them are provided in S1 Appendix.

In this study, we conducted two rounds of mixed-method community research to conceptualize COEN in urban China and to examine the psychometric properties of the COEN scale in real-world communities. Two components were included in the community research. First, we disseminated community surveys that included the COEN scale to residents who were diagnosed with hypertension and/or diabetes. To ensure sufficient sample size, the number of survey responses was at least 30 times the number of variables included in the factor analysis for the COEN scale [17]. Second, we conducted in-depth interviews with key informants in the local communities with respect to NCD management, to explore their perceptions about the concept of COEN and the scale, particularly regarding its relevance and comprehensibility, which further informed the modification of the scale items where necessary.

### Participants and sampling

The community research, including the community survey and key-informant interviews, was conducted in eastern China, in two selected cities (city S and city T) for the first round of research, and three (city S, city T, and city K) for the second round. These cities were purposively selected based on diversity in population sizes and urbanization levels, where city S was the most urbanized with the largest population, followed by city K, and city T had relatively the lowest urbanization level and the smallest population.

For community surveys, we recruited participants through stratified random sampling by residents’ sex and disease diagnosis (i.e. male and female, and hypertension and diabetes) from the electronic health record system of each community health center. The inclusion criteria for resident participants were: (1) aged 45 years or older; (2) having diagnosis of hypertension and/or diabetes from a certified health facility; (3) having lived in the community for at least nine months, with no plans to move out for at least another month; (4) able to provide informed consent. We excluded pregnant women, residents with terminal diseases, residents who were unable to communicate due to physical/mental disabilities.

For key-informant interviews, we included the following roles: (1) community residents, (2) community health providers, (3) officers of local township government, and (4) social workers. We excluded those who had been in these positions for less than six months, those who could not communicate with researchers, and those unable to provide informed consent. We conducted purposive sampling to identify eligible key informants and ensured sufficiency of sample size based on data saturation.

### Data collection

From 1^st^ to 30^th^ November 2018, we conducted the first round of community research. We disseminated questionnaires with the COEN scale and demographic information including gender, age, marital status, education level, and employment status. The COEN scale included all the items whose face validity was confirmed by the Delphi panels [16]. One month after the survey, we randomly selected 15% of the resident participants for the re-survey with the same questions to examine the test-retest reliability of the scale.

From 1^st^ July to 30^th^ August 2021, we conducted the second round of community research on a condensed version of the COEN scale, by selecting items with highest relevance and reported comprehensibility with linguistic modifications for improvements, which was informed by the first community research based on resident feedback and interviews. Similar data collection methods were used in this round, including a resurvey of 15% residents to examine test-retest reliability.

### Data analysis for surveys

For the first community survey in 2018, we first coded residents’ responses “don’t know” and “don’t want to reply” in the COEN items as missing values. We then excluded COEN items that had more than 10% missing values, which indicated the items’ lack of relevance and/or comprehensibility for respondents. After that, we performed Kappa test to examine the test-retest reliability for each item. Kappa values have the following meaning regarding test-retest reliability: <0.0 = poor; 0.00–0.20 = slight; 0.21–0.40 = fair; 0.41–0.60 = moderate; 0.61–0.80 = substantial; and 0.81–1.00 = almost perfect [18]. For items with Kappa values lower than 0.20, we re-considered their comprehensibility and relevance, and decided their exclusion or inclusion with. After that, we calculated the Cronbach’s alpha coefficient to examine the internal consistency of the COEN scale.

For the second community survey with the condensed COEN scale in 2021, we first conducted similar data analysis as in 2018, by identifying items with high numbers of missing values and low Kappa values. Then, we conducted exploratory factor analysis to determine the underlying multi-dimensionality of the COEN scale. To determine the number of factors to retain, we considered: (1) eigenvalues, where we kept factors whose eigenvalues were greater than 1.0 [19]; (2) parallel analysis, where we performed 100 iterations with randomly generated datasets in parallel with the actual dataset, and retained factors whose eigenvalues were greater than the randomly generated ones [20, 21]; and (3) scree plots: where we kept factors that did not pass the point on the scree plots in which the eigenvalues leveled off and began to form a straight line [22, 23]. Then, we used *oblimin* rotation for the factor analysis, to maximize the clarity of factor loadings while allowing different factors to correlate with each other [24]. An item was ascribed to a factor if its loading to the factor was 0.40 or higher. Items were considered for removal if they ascribed to none of the factors or to multiple factors (i.e. substantial cross-loadings). Finally, we calculated the Cronbach’s alpha coefficients to examine the internal consistency of the COEN scale and of each factor.

### Data analysis for interviews

We conducted content analysis on the key-informant interviews in both rounds of community research, focusing on two major topics. First, we explored interviewees’ understandings about key concepts, including community and COEN, to formalize our conceptualization of them. Second, we considered interviewees’ perceptions about the COEN scale items for inclusion, exclusion, and modification from their perspectives. For example, for items with suboptimal test-retest reliability in the community surveys, the interviews informed our decisions on whether to exclude those items or modify their wording to improve reliability. The final decision of keeping or removing each scale item was collectively informed by both the community surveys and key-informant interviews.

Two analysts (SX and GZ) performed survey data analysis independently, using Stata SE 15 (Stata Corp, College Station, TX, USA) and SPSS, respectively. Two analysts (SX and XC) led content analysis of interviews using NVivo 12 (QSR International).

### Ethics statement

The study was approved by the institutional review board of Duke Kunshan University (2018YAN003, 2019YANL013, and 2021YAN049). All participants provided written informed consent before being enrolled.

## Results

### Participant profile

In the first community research, we received 345 complete questionnaires, 120 from city S and 225 from city T (Table 1), with 99% response rate. The participants aged from 45–92 with a mean age of 67.4 (standard deviation = 8.2), and 50.1% were male. There were 80.6% participants diagnosed with hypertension and 46.4% diabetes, including 28.4% with both. Most residents were retired (89.0%), with 26.4% having primary school education or lower.

**Table 1:** Demographic information of survey participants in two rounds of community research.

	First community research (n = 345)	Second community research (n = 657)
Community ID (n, %)		
City S	120 (34.8%)	112 (17.1%)
City T	225 (65.2%)	216 (32.9%)
City K	0	329 (50.1%)
Age (mean, SD)	67.4, 8.2	66.3, 8.5
Gender (n, %)		
Male	173 (50.1%)	319 (48.5%)
Female	172 (49.9%)	338 (51.5%)
Hypertension (n, %)	278 (80.6%)	528 (80.4%)
Diabetes (n, %)	160 (46.4%)	372 (56.6%)
Employment status (n, %)		
Retired	307 (89.0%)	399 (60.7%)
Employed	22 (6.4%)	122 (18.6%)
Self-employed	6 (1.7%)	26 (4.0%)
Unemployed	10 (2.9%)	34 (5.2%)
Others*	0	76 (11.6%)
Marital status (n, %)		
Married	299 (86.7%)	602 (91.6%)
Spouse deceased	37 (10.7%)	40 (6.1%)
Divorced	5 (1.4%)	11 (1.7%)
Not married	4 (1.2%)	4 (0.6%)
Education level (n, %)		
≤ Primary school	91 (26.4%)	322 (49.0%)
Middle school	145 (42.0%)	211 (32.1%)
High school	88 (25.5%)	89 (13.6%)
≥ College	21 (6.1%)	35 (5.3%)

* Other employment status included freelancers, farmers, and veterans.

In the second community research, we received 657 complete questionnaires from city S (n = 112), city T (n = 216), and city K (n = 329) (Table 1), with 93% response rate. The participants had similar demographic profiles as the first community research, with fewer retired residents (60.7%) and more with primary school education or lower (49.0%).

For key-informant interviews, we conducted 27 in-depth interviews, including 21 community residents (two of them were residential committee representatives and two resident organization leaders), three community doctors, two social workers, and one township administrative officer.

### Conceptualizing “Community” and “Community Efficacy for NCD management”

We identified various distinctive understandings about the definition of “community” from the interviewees (Table 2, the upper half). For example, many residents considered their living neighborhood area as community; doctors in primary health care facilities considered the coverage of a community health center as community; and township government officers referred to community as their administrative reach. Deriving from these perspectives and anchored by NCD management, the present study conceptualized community as *“residents’ perceived routine living space*, *which includes their living neighborhood areas*, *residential committees*, *community health care facilities*, *civil societies*, *and government agencies”*.

**Table 2:** Conceptualizations for community and community efficacy for NCD management (COEN) based on key-informant interviews.

Concept	Conceptualization and representative quotes
**Community**	**A community refers to residents’ perceived routine living space, which includes their living neighborhood areas, residential committees, community health care facilities, civil societies, and government agencies.**
Representative quotes from interviews	*“To me*, *‘community’ is the neighborhood we live in every day*.*”* ––Resident C, diagnosed with hypertension
	*“In government documents*, *they sometimes refer to ‘community’ as a town*, *because that’s how far the governmental administration goes for resource allocation and supervision*.*”* ––Township government officer
	*“How to define the concept of ‘community’ depends on the perspective from which you look at it*. *For NCD management in urban China*, *a community could be ‘a community health station’s coverage of residential area’*, *because the stations are where people get community health services*. *Having said that*, *the coverage of residential committee could also be a plausible definition*, *because that’s the basic unit for most community activities*.*”* ––Expert T in community health management
**Community efficacy for NCD management**	**COEN refers to the ability of a community to provide NCD management for its residents, reflected by its natural environment, social relationships, community resources, health services, and resident-engaging activities.**
Representative quotes from interviews	*“The management of NCDs at the community level in China must consider the community health centers and stations*. *They are required to provide National Essential Public Health Services*, *including those for hypertension and diabetes*, *for example*.*”* ––Expert C in health promotion, male
	*“People*, *especially the older adults who might be hard to travel*, *depend on communities*. *So it is important and obligated for communities to have strong capacity to manage NCDs*.*”* ––Community health provider L, general practitioner
	*“As social workers*, *our major responsibility is to engage the residents through community activities*, *like festival events or daily dancing groups*. *These things could make them happy and healthy*, *especially for those who live alone*.*”* ––Social worker W of local civil society
	*“In many urbanized communities*, *they already have almost everything*: *facilities*, *activities*, *services…You know what they lack*? *The mobilization of all these things to serve the well-being of the residents*. *This needs to be done and should be done properly*.*”* ––Expert G in community health management

The COEN construct was also enriched by community research (Table 2, the lower half). Some interviewees mentioned community-based health services as an important component of COEN. A social worker added the importance of resident engagement in community activities, such as peer-support groups and festival events. One expert further emphasized the mobilization of resources to serve residents’ well-being as an important aspect of COEN. In summary, we conceptualized COEN as *“the ability of a community to provide NCD management for its residents*, *reflected by its natural environment*, *social relationships*, *community resources*, *health services*, *and resident-engaging activities*.*”*

### First community research

The first community research included all 71 potential items of the COEN scale from the Delphi panels, and they were divided into five groups based on the meaning of the items (Table 3), including items about community physical environment, NCD behavioral risk factors, resident mental health and social relationships, community health management, and community organizations and activities. Of the 71 items, six were removed due to a high percentage (≥ 10%) missing values, and another 21 were removed due to low Kappa values (< 0.20). We further removed six items due to incomprehensibility reported in key-informant interviews. Notably, seven items were kept despite the suboptimal Kappa values because of their theoretical relevance to COEN, and they were linguistically modified to increase the clarity and comprehensibility. Details about these modifications were included in S1 Table.

**Table 3:** Results from the first community research on the “community efficacy for non-communicable disease management” (COEN) scale.

	Original number of items	Number of items removed due to high numbers of missing values (n, %)	Number of items removed due to low kappa values (n, %)	Number of items whose removal was informed by interviews (n, %)	Number of items kept without modifications (n, %)	Number of items kept & modified, informed by interviews (n, %)	Cronbach’s alpha among kept items
Group 1: Community physical environment	13	1 (8%)	3 (23%)	0	9 (69%)	0	0.80
Group 2: NCD behavioral risk factors	19	2 (11%)	4 (21%)	2 (11%)	10 (53%)	1 (5%)	0.56
Group 3: Mental health and social relationships	12	0	4 (33%)	2 (17%)	5 (42%)	1 (8%)	0.61
Group 4: Community health management	13	0	6 (46%)	2 (15%)	2 (15%)	3 (23%)	0.72
Group 5: Community organizations and activities	14	3 (21%)	4 (29%)	0	4 (29%)	3 (21%)	0.78
Overall	71	6 (8%)	21 (30%)	6 (8%)	30 (42%)	8 (11%)	0.86

With the 38 remaining items, the overall Cronbach’s alpha coefficient was 0.86 (Table 3). All separate groups of items had a Cronbach’s alpha coefficient ≥ 0.70, except for the group of items about NCD behavioral risk factors (alpha = 0.56) and the group about mental health and social relationships (alpha = 0.61), which still exceeded the “acceptable internal consistency” threshold of 0.60.

### Second community research

Grounded by the first community research, the second round of community research used a condensed version of the COEN scale with 19 items of highest reported relevance and comprehensibility (Table 4). None of the 19 items was excluded because of high numbers of missing values in the survey. In the factor analysis, the eigenvalues, scree plots (S1 Fig), and parallel analysis (S2 Fig) all suggested the retention of five factors. After *oblimin* rotation, the highest loading was higher than 0.40 for each item. We observed substantial cross-loading only in item #18, where the loadings for Factor One and Five were 0.54 and 0.44 respectively. Collectively, the five factors explained 64.23% of the variance. A complete final 14-item COEN scale is provided in S2 Appendix.

**Table 4:** Exploratory factor analysis for second validation research in 2021*.

COEN scale items	Kappa, P value	Factor One	Factor Two	Factor Three	Factor Four	Factor Five	Uniqueness	Decision
1. How is the sanitary condition of the public areas in your community?	0.21, 0.01	**0.65**			0.26		0.45	Keep
2. How safe do you feel, living in your community (e.g. security, road safety)	0.14, 0.07**	**0.49**			0.27		0.52	Remove
3. How is the availability of public facilities in your community? (e.g. parks, gyms)	0.41, < 0.01	**0.67**					0.51	Keep
4. How convenient is the transportation in your community for you to get around?	0.45, < 0.01	0.35		**0.57**			0.41	Keep
5. How accessible are fresh fruits and vegetables in your community?	0.28, < 0.01			**0.89**			0.21	Keep
6. How easy is it for you to buy alcohol and tobacco products in your community?	0.31, < 0.01			**0.88**			0.25	Keep
7. How severe is the second-hand smoking issue in your community	0.19, 0.03**			0.23	**-0.53**		0.68	Remove
8. How often do you engage in physical activities with people in your community?	0.39, < 0.01		**0.69**			0.21	0.46	Keep
9. How often do you interact with other residents? (e.g. sharing news, helping out)	0.47, < 0.01		**0.78**				0.38	Keep
10. How much do you trust other residents in your community?	0.26, < 0.01		**0.65**		0.27		0.42	Keep
11. How many friends do you have in your community?	0.42, < 0.01	0.22	**0.67**				0.43	Keep
12. How is the general mental health status among people in your community?	0.16, 0.05**	0.29	**0.46**		0.25		0.54	Remove
13. How well do the community health services meet your regular health needs?	0.24, < 0.01				**0.65**		0.53	Keep
14. How satisfied are you with the community health services?	0.04, 0.38**			0.21	**0.60**		0.52	Remove
15. How do you think of the cost of community health services, considering quality?	0.41, < 0.01			0.21	**0.57**	0.21	0.59	Keep
16. How abundant were community activities in your community in the past year?	0.23, 0.01	**0.61**				0.39	0.41	Keep
17. How involved were you in these community activities in the past year?	0.42, < 0.01					**0.78**	0.39	Keep
18. How abundant are resident organizations in your community?	0.17, 0.04**	**0.54**				**0.44**	0.41	Remove
19. How involved were you in resident organizations in the past year?	0.51, < 0.01					**0.80**	0.33	Keep

* Factor loadings lower than 0.20 were suppressed; boldness indicated factor loadings ≥ 0.40.

** Marked items whose kappa values were lower than 0.20.

Factor One included five items, with an eigenvalue of 2.89 (Table 4). With item #18 removed due to substantial cross-loadings and item #2 removed for low test-retest reliability (Kappa = 0.14, P = 0.07), three items in this factor remained, which focused on “community management” from the perspective of sanitation, public facilities, and organizations of community activities. Another five items ascribed to Factor Two with an eigenvalue of 2.75. With item #12 removed due to low test-retest reliability (Kappa = 0.16, P = 0.05), the remaining four items in this factor focused on residents’ “social relationships” such as interactions and friendships in the community. Factor Three included three items with an eigenvalue of 2.4, which focused on residents’ “accessibility” to community resources, such as public transportation and commodities. Another three items ascribed to Factor Four with an eigenvalue of 2.17. With item #14 removed for low test-retest reliability (Kappa = 0.04, P = 0.38), the remaining two items in this factor focused on the availability and costs of “community health services”. Finally, three items ascribed to Factor Five, with an eigenvalue of 1.97. After removing item #18 for substantial cross-loadings, the two remaining items focused on residents’ “engagement” in community activities and organizations.

The overall Cronbach’s alpha coefficient for the COEN scale was 0.79. Within each of the five factors, the Cronbach’s alpha coefficients were 0.60, 0.71, 0.75, 0.42, and 0.71, respectively. No further removal of any items increased the Cronbach’s alpha coefficients of the overall scale or within each factor.

In summary, the final validated condensed COEN scale included a total of 14 items covering five factors: community management (number of items = 3), social relationships (n = 4), resource accessibility (n = 3), community health services (n = 2), and resident engagement (n = 2).

## Discussion

In this study, we developed the concept of COEN, denoting the ability of a community to provide NCD management for residents. Although community-based NCD management is not new to the public health literature, our conceptualization of COEN with measurement scale provides an innovative contribution. Through a three-step process including two rounds of validation research in China, we finalized a condensed 14-item COEN scale, comprised of five dimensions: community management, social relationships, resource accessibility, community health services, and resident engagement. The study ensured the reliability (i.e. internal consistency and test-retest reliability) and validity (i.e. face and structural validity) of the scale, a strength many scale validation studies do not possess [25].

Although COEN specifically focuses on community-based NCD management, it also corresponds to existing concepts and theories in the literature. “Community mobilization”, for example, is associated with positive behavioral change and health outcomes [12]. Although the definition of community mobilization is not consistent across studies, its generic connotation as “a capacity building process of community members” is highly compatible with COEN, where high COEN should enable successful community mobilization through optimal community management, social relationships, and resident engagement. Second, “community engagement” emphasizes the involvement of community members in implementing specific interventions or one-off programs [14]. This is distinct from COEN because the latter is not program-driven but rather a consistent property of the community related with NCD management. However, community engagement as a program-driven strategy is believed to strengthen community-based NCD management [14], which, in our language, means the improvement of COEN. Finally, “community empowerment” is most closely related to COEN. Laverack *et al*. 2006 mentioned community empowerment as a means to attaining power to communities to improve lives and health, which entails nine domains including community-based organizations, local leadership, and resource mobilization [13]. These domains strongly correspond to the five dimensions of COEN. However, their concept remains focused on the “process”, while COEN focuses on the “status” of communities under observation. Hence, from the perspective of NCD management, community empowerment could be considered as the process of improving COEN, and improved COEN the desired outcome of community empowerment. In summary, a key advantage of COEN is its theoretical novelty, which provides a new and comprehensive perspective to community-based NCD management, but it is also grounded by the existing concepts and theories in community-based research with prominent associations.

Many measurements of related concepts were developed prior to our study. A systematic review on empowerment measures for health promotion identified 20 scales, but most of them focused on individuals, such as social workers’ professional power and patients’ perceptions of provider support [25]. Of note, one study conducted in Estonia used an “individual community-related empowerment” scale to assess the effects of community health interventions. Their 18-item scale represented residents’ confidence in their personal capacities, willingness, sense of importance, and sense of responsibility to be involved in community actions of common goals. This scale, although community-oriented, still focused on individual residents’ qualities [26]. The COEN scale, on the other hand, measures the qualities of communities as perceived by residents. It fills a gap among existing scales that lack quantified measurements at the community level [25].

We envision four major uses for the COEN scale. First, at the individual level, community workers and practitioners are encouraged to use it to identify residents with negative perceptions of the community, which may signal their loneliness and social isolation, especially in the “social relationships” and “resident engagement” dimensions. Social isolation was found to be associated with increased risk of mortality among community-dwelling older adults [27]. Using the COEN scale for this purpose should be done with caution to protect residents’ privacy, and is recommended to be combined with other psychological measures such as depression and loneliness scales. Second, at the community level, by collecting a group of responses, the COEN scale could support the examination of communities’ strengths and weaknesses in NCD management. Researchers are encouraged to utilize the scale for process evaluation and/or outcome assessment for community-based NCD interventions, in addition to traditional measurements such as biological and behavioral indicators. Third, local health administrators may use the COEN scale to conduct cross-sectional comparisons across different communities to generate insights for local health governance. Finally, global health practitioners, researchers, as well as policy makers could also use the COEN scale to conduct longitudinal assessment of community performances in NCD management over time, which could potentially inform the development of intervention strategies tailored to local community contexts. Notably, we recommend the condensed 14-item version of the COEN scale due to the ease of dissemination and scalability. The 38-item version is suitable when deeper investigations about particular aspects of COEN are needed.

A key strength for the development of the COEN concept and scale was the adoption of a three-step approach, which leveraged multiple scientific methodologies including the previously reported literature review and Delphi panels, and the community research described in the present study, strengthened by the meta-inference based on both quantitative and qualitative data. Several limitations of this study should also be acknowledged. First, our sampling strategy for community residents through electronic record systems limited the external validity of our scale because we were not able to capture residents who did not interact with community health facilities. We compensated for such potential loss of data by interviewing community key-informants familiar with the general situation of the communities. Second, questions about respondents’ experience in the communities might be subject to social desirability bias. To reduce this concern, we trained researchers to inform respondents of the impartial nature of the study and the importance of authentic responses. Third, the communities included for validation research were restricted to urban settings in eastern China, which may limit the generalizability of the findings. Local customization and validation are thus recommended when using the scale in other locations.

## Conclusions

The concept and measurement scale of COEN can be used to assist future efforts for NCD management anchored in the community. Community leaders, health administrators, and decision makers could use COEN to identify local priorities, plan and evaluate community-based NCD management interventions. The study also provides three implications for future research. First, more empirical research is needed to explore the associations between the COEN scale and people’s health outcomes, to further establish causal pathways in community-based NCD management. Second, studies to assess the usability of the COEN scale beyond urban settings in China could contribute to increasing the generalizability of the findings and the validity of COEN as a universal tool. Finally, extending the COEN concept for other health issues, such as infectious diseases and maternal and child health [14, 28], is also worth exploring.

## Supporting information

S1 Checklist Inclusivity in global research. (DOCX)

S1 Appendix Literature review, expert interviews and Delphi panels for scale development. (DOCX)

S2 Appendix Community efficacy for noncommunicable disease management scale (COEN-14). (DOCX)

S1 Table Detailed results from 2018 validation research on the “community efficacy for non-communicable disease management” (COEN) scale. (DOCX)

S1 Fig Scree plot of eigenvalues in exploratory factor analysis. (DOCX)

S2 Fig Results from the parallel analysis with 100 iterations for exploratory factor analysis. (DOCX)
