# Supplementary material for: Community efficacy for non-communicable disease management (COEN): Conceptualization and measurement
Source: PLOS Glob Public Health. 2024 Aug 14;4(8):e0003549. doi: 10.1371/journal.pgph.0003549 (PMC11324141; doi:10.1371/journal.pgph.0003549)
Supplement: S1 Appendix — (DOCX) [file pgph.0003549.s002.docx]

**S1 Appendix. Literature review, expert interviews and Delphi panels for scale development**

**Literature review and expert interviews**

We first conducted a literature review on the topic of community-based NCD management, to explore existing evidence, practices, and theories about community based NCD globally and in China. We then conducted unstructured individual interviews with experts in academia and community organizations in the field of community health, NCD management, and health promotion, which informed the initial conception of our key research questions and concepts.

**Delphi panels for scale development**

We then conducted two rounds of Delphi panels, involving 30 experts from China and beyond, with at least 10-year expertise in community health, NCD management, and health promotion. The Delphi panels reached consensus in the selection of COEN scale items and further informed the structure of the COEN scale with high face validity.

**Initial structure of COEN scale**

These two steps yielded a total of 71 items for the COEN scale with consistency in the high face validity. Each item represented a question to be answered by the community residents on a five-level Likert scale (e.g. very good, good, normal, bad, and very bad). The Delphi panels further structured the COEN items into five groups based on the meaning of each item:

First, the “*community physical environment*” group included items that focused on pollution, safety, and availability of public facilities. These items were agreed by the Delphi panels to either have direct effects on residents’ NCD management or could affect residents desires to pursue healthy lifestyles.

Second, the “*community-level behavioral risk factors*” included items that focused on tobacco use, alcohol use, physical activity, dietary behaviors, and health literacy levels in the community, which was widely believed to be associated with NCD management.

Third, the “*community mental health & social relationships*” group included items that reflected community residents’ mental health status and their level of cohesion and peer-support among residents, which were agreed by the experts to play important roles in NCD management.

Fourth, the “*community health management*” group included items that measured the availability, quality, and affordability of health resources in the community with a focus on NCD management.

Finally, the “*community activities & organizations*” group included items that focused on the abundancy, helpfulness, and residents’ engagement in community-based activities and organizations, which the Delphi experts agreed could have indirect influence on residents’ NCD management through information exchange and behavioral changes.

**Qualitative perceptions about COEN scale**

Most experts reported that the COEN scale was a comprehensive tool to examine residents’ perceptions about their communities’ abilities to NCD management under a reasonable structure. The majority of the participants found the questions to be understandable, with a few issues brought up about the accessibility for older adults.

*“The intention of each question is clear to me, but for some older folks, it might be less easy. Try to rephrase some questions to be friendlier for less educated older adults, especially about those ‘community organizations and activities’”*

–Township Administrative Officer W

Some community residential committee workers found the scale useful for their routine responsibilities:

“*In some communities, there are many people who came from other cities or provinces for work, and do not interact much with others and that may become an issue. The ‘mental health and social relationship’ part, in particular, may help us (residential committee) identify such people.*”

–Residential Committee Worker L

One fieldwork researcher mentioned the likelihood of social desirability bias and power dynamics when disseminating the COEN scale.

*“Some people, especially the elderly, might be subject to giving all good answers in the questionnaire just because they wanted to be in favor of the community leaders or doctors, or in fear of losing their favor. That’s why researchers should tell residents that this is anonymous and the purpose of the questionnaire is to help them identify areas for improvements in the community.”*

–Fieldwork Researcher M
