# Supplementary material for: Community efficacy for non-communicable disease management (COEN): Conceptualization and measurement
Source: PLOS Glob Public Health. 2024 Aug 14;4(8):e0003549. doi: 10.1371/journal.pgph.0003549 (PMC11324141; doi:10.1371/journal.pgph.0003549)
Supplement: S2 Appendix — (DOCX) [file pgph.0003549.s003.docx]

**S2 Appendix.** **Community Efficacy for Noncommunicable Disease Management Scale (COEN-14)**

1. How is the sanitary condition of the public areas in your community? (Please consider littering, garbage recycling, pet waste, etc.)

a. Very clean b. Clean c. Fair d. Dirty e. Very dirty

1. How is the availability of public facilities in your community? (Please consider greenway, parks, playground, facilities for physical activities, etc.)

a. Very good b. Good c. Fair d. Bad e. Very bad

1. How convenient is the transportation in your community for you to get around? (Please consider the accessibility of public transportation/ hospital/schools/grocery stores, etc.)

a. Very convenient b. Convenient c. Fair d. Inconvenient e. Very inconvenient

1. How accessible are fresh fruits and vegetables in your community?

a. Very convenient b. Convenient c. Fair d. Inconvenient e. Very inconvenient

1. How easy is it for you to buy alcohol and tobacco products in your community?

a. Very convenient b. Convenient c. Fair d. Inconvenient e. Very inconvenient

1. How often do you engage in physical activities with people in your community?

a. Always b. Often c. Sometimes d. Occasionally e. Almost never

1. How often do you interact with other residents in your community? (Such as offering or receiving help, hanging out together, small talks, sharing information and news, etc.)

a. Always b. Often c. Sometimes d. Occasionally e. Almost never

1. How much do you trust other residents in your community?

a. Very trusting b. Somewhat trusting c. Fairly d. Somewhat distrustful e. Very distrustful

1. How many friends do you have in your community?

a. A lot b. Many c. Average d. Not much e. Few

1. How well do the *community health services* meet your regular health needs? (Please consider the availability of consultation, meditations, and health check services that you need from the most accessible community health facility, without turning to higher-level hospitals.)

a. Very sufficient b. Sufficient c. Fair d. Insufficient e. Very insufficient

1. How do you think of the cost of the *community health services*? (Please take into account the service quality.)

a. Very reasonable b. Reasonable c. Fair d. Unreasonable e. Very unreasonable

1. In the past year, how abundant were *community activities* in your community (Please consider activities initiated by government agencies, residential committees, community health facilities, and civil societies, including but not limited to celebration events, charity events, festival events, sports competitions, etc.)

a. Very abundant b. Abundant c. Fair d. Rare e. Very rare

1. In the past year, how involved were you in these *community activities*?

a. Highly involved, sometimes as organizer/volunteer b. Highly involved but only as participants

c. Much involved d. Sometimes involved e. Seldom or never involved

1. In the past year, how involved were you in *resident organizations*? (Please consider your engagement with organizations organized among by residents, including but not limited to interest groups such as square dance clubs, reading club, and peer-support groups such as hypertension self-management groups.)

a. Highly involved, sometimes as organizer/volunteer b. Highly involved but only as member

c. Much involved d. Sometimes involved e. Seldom or never involved
