# Supplementary material for: Community efficacy for non-communicable disease management (COEN): Conceptualization and measurement
Source: PLOS Glob Public Health. 2024 Aug 14;4(8):e0003549. doi: 10.1371/journal.pgph.0003549 (PMC11324141; doi:10.1371/journal.pgph.0003549)
Supplement: S1 Table — (DOCX) [file pgph.0003549.s004.docx]

**S1 Table. Detailed results from 2018 validation research on the “community efficacy for non-communicable disease management” (COEN) scale**

| Scale items* | Missing value  (N, %) | Kappa and P value | Decision code** |
| --- | --- | --- | --- |
| **Group 1: Community physical environment** (Cronbach’s alpha: 0.80) |  |  |  |
| 1. How is the level of noise in your community? | 1, 0.3% | 0.14, 0.09 | Removal-B |
| 2. How clean is your community's water for residents, such as tap water? | 3, 0.9% | 0.19, 0.04 | Removal-B |
| 3. How clean are the public water sources, such as lakes and streams, in your community? | 60, 17.4% | 0.12, 0.13 | Removal-A |
| 4. How is the air quality in your community? | 1, 0.3% | 0.42, <0.01 | Keep-A |
| 5. How is the sanitary condition of the public areas in your community? | 1, 0.3% | 0.52, <0.01 | Keep-A |
| 6. How is the level of light pollution in your community? | 3, 0.9% | 0.44, <0.01 | Keep-A |
| 7. How safe is the building environment in your community? | 3, 0.9% | 0.20, 0.02 | Keep-A |
| 8. How is the road safety in your community? | 1, 0.3% | 0.26, <0.01 | Keep-A |
| 9. How much violent and destructive behavior is there in your community? | 3, 0.9% | 0.17, 0.13 | Removal-B |
| 10. How is the security system in your community? | 5, 1.4% | 0.26, <0.01 | Keep-A |
| 11. How is the availability of public facilities in your community? | 4, 1.2% | 0.33, <0.01 | Keep-A |
| 12. How convenient is the transportation in your community for you to get around? | 0 | 0.20, 0.02 | Keep-A |
| 13. How do you consider the overall habitability in your community? | 0 | 0.25, <0.01 | Keep-A |
| **Group 2: NCD behavioral risk factors** (Cronbach’s alpha: 0.56) |  |  |  |
| 14. How easy is it for you to buy tobacco products in your community? | 23, 6.7% | 0.31, 0.03 | Keep-B |
| 15. How is the regulation for public area smoking in your community? | 29, 8.4% | 0.34, <0.01 | Keep-A |
| 16. How prevalent is tobacco use in your community? | 15, 4.3% | 0.31, <0.01 | Keep-A |
| 17. How severe is the second-hand smoking issue in your community? | 13, 3.8% | 0.22, 0.02 | Keep-A |
| 18. How often do you get handed cigarettes or invited to smoke by other residents? | 8, 2.3% | 0.27, 0.01 | Removal-C |
| 19. How convenient is it to buy alcohol in or near your community? | 12, 3.5% | 0.16, 0.06 | Removal-B |
| 20. How prevalent is alcohol consumption in your community? | 62, 18.0% | 0.12, 0.17 | Removal-A |
| 21. How often do you receive alcohol or get invited to drink alcohol by other residents? | 17, 4.9% | 0.38, <0.01 | Removal-C |
| 22. How is the space for physical activity in your community? | 6, 1.7% | 0.11,0.15 | Removal-B |
| 23. How active are people in your community in terms of exercise/physical activities? | 10, 2.9% | 0.33, <0.01 | Keep-A |
| 24. How often do you engage in physical activities with people in your community? | 1, 0.3% | 0.40, <0.01 | Keep-A |
| 25. How is the food safety in your community? | 20, 5.8% | 0.32, <0.01 | Keep-A |
| 26. How accessible are fresh fruits and vegetables in your community? | 2, 0.6% | 0.26, <0.01 | Keep-A |
| 27. How healthy are the diet habits of residents in your community? | 37, 10.7% | 0.14, 0.20 | Removal-A |
| 28. How often are you offered fresh fruits or vegetables by other residents? | 10, 2.9% | 0.10, 0.20 | Removal-B |
| 29. How is the level of health knowledge of residents in your community? | 15, 4.3% | 0.12, 0.15 | Removal-B |
| 30. How are the residents’ attitude towards pursuing healthy life in your community? | 9, 2.6% | 0.22, <0.01 | Keep-A |
| 31. How healthy are residents’ behavior in general in your community? | 9, 2.6% | 0.30, <0.01 | Keep-A |
| 32. How are residents’ abilities to access and apply health knowledge in your community? | 23, 6.7% | 0.28, <0.01 | Keep-A |
| **Group 3: Mental health and social relationships** (Cronbach’s alpha: 0.61) |  |  |  |
| 33. How prevalent are mental health issues (e.g. depression, anxiety) in your community? | 17, 4.9% | 0.36, <0.01 | Keep-A |
| 34. How severe is mental stress among residents in your community? | 24, 7.0% | 0.39, <0.01 | Removal-C |
| 35. How is residents’ happiness in your community? | 3, 0.9% | 0.33, <0.01 | Keep-A |
| 36. How is the sufficiency of mental health resources in your community? | 24, 7.0% | 0.22, 0.03 | Keep-A |
| 37. To what extent are the above mental health resources accepted or used by residents? | 25, 7.2% | 0.35, <0.01 | Removal-C |
| 38. How well do you know other community residents? | 1, 0.3% | 0.11, 0.15 | Removal-B |
| 39. How often do residents communicate with each other in your community? | 4, 1.2% | 0.16, 0.07 | Removal-B |
| 40. How many friends do you have in your community? | 1, 0.3% | 0.22, 0.01 | Keep-A |
| 41. How much do you trust other people in your community? | 3, 0.9% | 0.24, 0.02 | Keep-A |
| 42. How often do residents provide material support to each other in your community? | 7, 2.0% | 0.17, 0.04 | Removal-B |
| 43. How often do residents provide emotional support to each other in your community? | 5, 1.4% | 0.03, 0.36 | Removal-B |
| 44. How often do residents interact with each other in your community? | 4,1.2% | 0.37, <0.01 | Keep-B |
| **Group 4: Community health management** (Cronbach’s alpha: 0.72) |  |  |  |
| 45. How well do you know about national essential public health services? | 5, 1.4% | 0.23, 0.02 | Removal-C |
| 46. How do you think about the health education provided in your community? | 15, 4.3% | 0.07, 0.22 | Removal-B |
| 47. How is the public health service regarding hypertension in your community? | 6, 1.7% | 0.28, <0.01 | Keep-A |
| 48. How is the public health service regarding diabetes in your community? | 24, 7.0% | 0.22, <0.01 | Keep-A |
| 49. How sufficient are the health services provided by your community? | 16, 4.6% | 0.05, 0.31 | Keep-B |
| 50. How is the quality of health services provided by your community? | 9, 2.6% | 0.26, <0.01 | Keep-B |
| 51. How convenient could you use health services in your community? | 6, 1.7% | 0.15, 0.05 | Removal-B |
| 52. How do you think of the price of health services in your community? | 19, 5.5% | 0.19, 0.03 | Keep-B |
| 53. How is the acceptability of health services in your community? | 4, 1.2% | -0.04, 0.67 | Removal-B |
| 54. How well do you know about NCD issues in your community? | 14, 4.1% | -0.08, 0.75 | Removal-B |
| 55. How important do you think NCD issues are in your community? | 3, 0.9% | 0.02, 0.44 | Removal-B |
| 56. How involved are the residents in NCD management in your community? | 4, 1.2% | 0.06, 0.29 | Removal-B |
| 57. How well is your community mobilizing residents to address NCD issues? | 22, 6.4% | 0.22, 0.02 | Removal-C |
| **Group 5: Community organizations and activities** (Cronbach’s alpha: 0.78) |  |  |  |
| 58. How much does your community administration value residents’ involvement? | 23, 6.7% | 0.26, <0.01 | Keep-A |
| 59. How involved are residents in the decision-making in your community? | 26, 7.5% | 0.01, 0.48 | Removal-B |
| 60. How well is your community encouraging residents to participate in decision-making? | 42, 12.2% | 0.10, 0.18 | Removal-A |
| 61. How abundant are the resident organizations in your community? | 14, 4.1% | 0.28, <0.01 | Keep-A |
| 62. How helpful are those resident organizations for residents? | 20, 5.8% | 0.16, 0.08 | Removal-B |
| 63. How often do residents participant in those organizations? | 19, 5.5% | 0.16, 0.07 | Keep-B |
| 64. How is residents’ experience in participating in resident organizations? | 38, 11.0% | 0.15, 0.08 | Removal-A |
| 65. How is the abundancy of civil society organizations in your community? | 40, 11.6% | 0.03, 0.39 | Removal-A |
| 66. How helpful are those civil society organizations for residents? | 28, 8.1% | 0.08, 0.23 | Removal-B |
| 67. How is the abundancy and frequency of activities in your community? | 21, 6.1% | 0.03, 0.39 | Keep-B |
| 68. How well are those community activities organized and managed? | 24, 7.0% | 0.22, 0.02 | Keep-A |
| 69. How helpful are those community activities for residents? | 12, 3.5% | 0.23, 0.02 | Keep-A |
| 70. How involved were the residents in community activities in the past year? | 19, 5.5% | 0.05, 0.32 | Keep-B |
| 71. How are residents’ experiences in those community activities? | 27, 7.8% | 0.06, 0.35 | Removal-B |

** For item #44, #63, and #70, which asked about the level of interaction among community residents and their involvement in resident organizations and community activities, respondents suggested to change the question subject from “other residents” to the respondents themselves. For example, “How often do residents in your community interact with each other” was changed into “How often do you interact with other residents in your community”. For item #49, #50, and #52, which asked about residents’ perceptions about community health services, it was suggested to change the wording to better fit the residents’ perspective. For example, item #50 was changed from “How is the quality of the community health services” into “How satisfied are you with the community health services”. For item #67, which asked about the abundancy of community activities, it was suggested to add more explanations with specific examples about what community activities were. For item #14, “How convenient is it to buy tobacco products in your community”, it was suggested to add “alcohol” to the question due to the common combination of tobacco and alcohol in the community stores.*

*** For the decision codes, “Keep-A” means to keep this item without modifications. “Keep-B” means to keep this item (sometimes despite of its low Kappa values) with modifications informed by the interviews and/or focus group discussions. “Removal-A” means removal due to high percentages of missing values (>10%). “Removal-B” means removal due to low Kappa values (<.020). “Removal-C” means removal informed by the interviews and/or focus group discussions.*
