# Supplementary material for: Community efficacy for non-communicable disease management (COEN): Conceptualization and measurement
Source: PLOS Glob Public Health. 2024 Aug 14;4(8):e0003549. doi: 10.1371/journal.pgph.0003549 (PMC11324141; doi:10.1371/journal.pgph.0003549)
Supplement: S1 Fig — (DOCX) [file pgph.0003549.s005.docx]

**S1 Fig. Scree plot of eigenvalues in exploratory factor analysis***


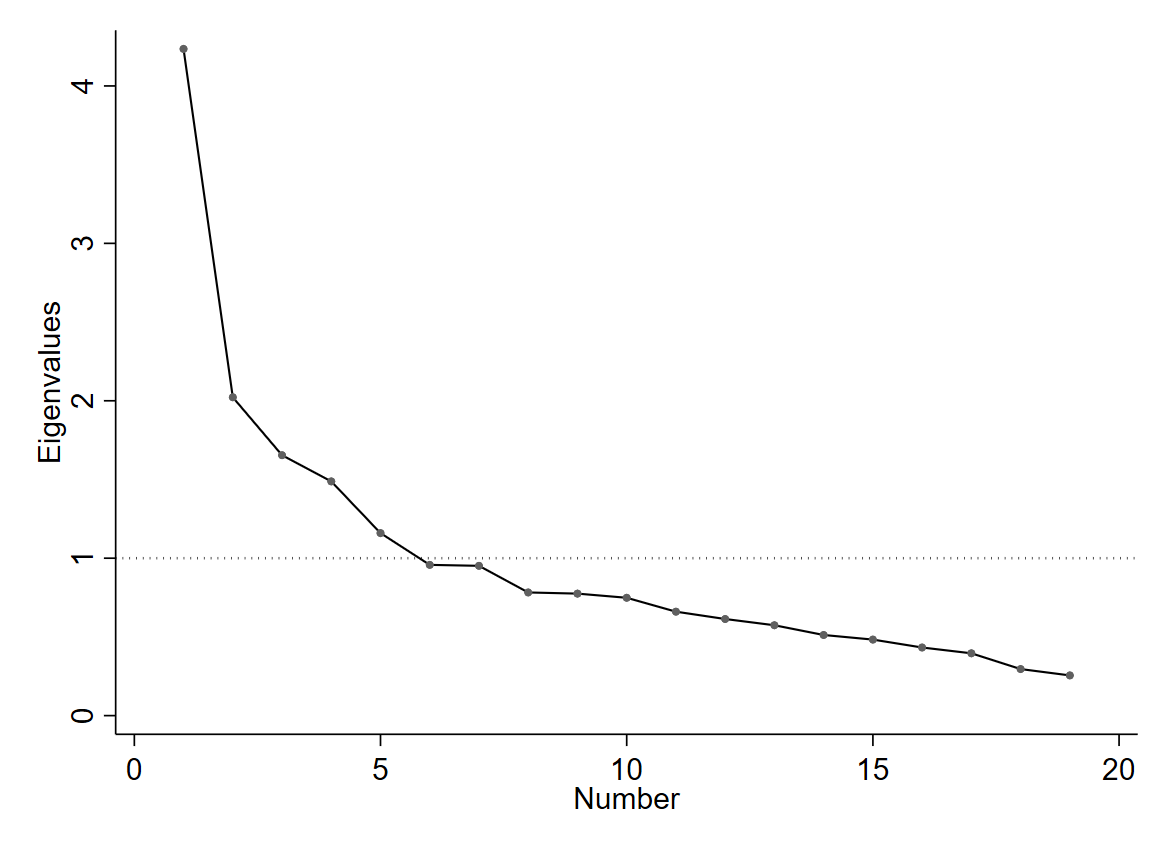


* The Eigenvalues were above one for the first five factors and went under after that, which formed a fairly straight line.
