# Supplementary material for: Community efficacy for non-communicable disease management (COEN): Conceptualization and measurement
Source: PLOS Glob Public Health. 2024 Aug 14;4(8):e0003549. doi: 10.1371/journal.pgph.0003549 (PMC11324141; doi:10.1371/journal.pgph.0003549)
Supplement: S2 Fig — (DOCX) [file pgph.0003549.s006.docx]

**
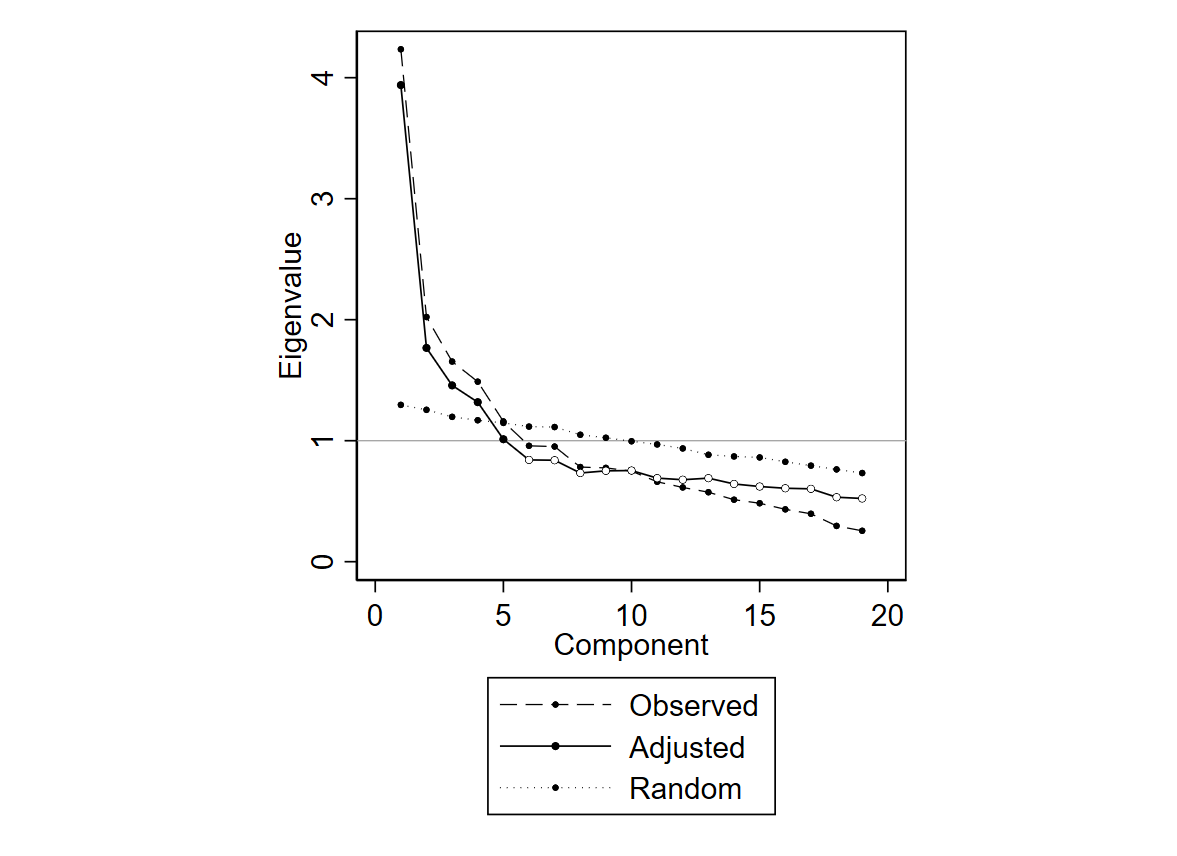
S2 Fig. Results from the parallel analysis with 100 iterations for exploratory factor analysis***

* The Eigenvalues for the observed and random lines met at five on the X axis.
